# Genome-wide association analysis identified splicing single nucleotide polymorphism in CFLAR predictive of triptolide chemo-sensitivity

**DOI:** 10.1186/s12864-015-1614-1

**Published:** 2015-06-30

**Authors:** Lata Chauhan, Gregory D. Jenkins, Neha Bhise, Tanya Feldberg, Taraswi Mitra-Ghosh, Brooke L. Fridley, Jatinder K. Lamba

**Affiliations:** Department of Pharmacotherapy and Translational Research, University of Florida, Gainesville, FL USA; Department of Health Sciences Research, Mayo Clinic, Rochester, MN USA; Department of Experimental and Clinical Pharmacology, University of Minnesota, Minneapolis, MN USA; Department of Biostatistics, University of Kansas Medical Center, Kansas City, KS USA

**Keywords:** Triptolide, Genome-wide association studies, Hap-map, Single nucleotide polymorphisms, CFLAR

## Abstract

**Background:**

Triptolide is a therapeutic diterpenoid derived from the Chinese herb *Tripterygium wilfordii Hook f*. Triptolide has been shown to induce apoptosis by activation of pro-apoptotic proteins, inhibiting NFkB and c-KIT pathways, suppressing the Jak2 transcription, activating MAPK8/JNK signaling and modulating the heat shock responses.

**Results:**

In the present study, we used lymphoblast cell lines (LCLs) derived from 55 unrelated Caucasian subjects to identify genetic markers predictive of cellular sensitivity to triptolide using genome wide association study. Our results identified SNPs on chromosome 2 associated with triptolide IC_50_ (p < 0.0001). This region included biologically interesting genes as CFLAR, PPIl3, Caspase 8/10, NFkB and STAT6. Identification of a splicing-SNP rs10190751, which regulates CFLAR alternatively spliced isoforms predictive of the triptolide cytotoxicity suggests its role in triptolides action. Our results from functional studies in Panc-1 cell lines further demonstrate potential role of CFLAR in triptolide toxicity. Analysis of gene-expression with cytotoxicity identified JAK1 expression to be a significant predictor of triptolide sensitivity.

**Conclusions:**

Overall out results identified genetic factors associated with triptolide chemo-sensitivity thereby opening up opportunities to better understand its mechanism of action as well as utilize these biomarkers to predict therapeutic response in patients.

**Electronic supplementary material:**

The online version of this article (doi:10.1186/s12864-015-1614-1) contains supplementary material, which is available to authorized users.

## Background

Triptolide is a biological diterpenoid derived from the Chinese herb *Tripterygium wilfordii HOOK f*. Triptolide has been shown to have anti-inflammatory and immunosuppressive activities and has been used in traditional Chinese medicine to treat several diseases, such as, rheumatoid arthritis, immune complex diseases, and systemic lupus erythematosus [1, 2]. It has been shown to have influence on several anti-tumor target genes and inhibit tumors by altering multiple signaling pathways, such as, inhibition of NFκB and c-KIT pathway [3], inhibition of Jak2 transcription [4], inducing apoptotic signals by activation of pro-apoptotic proteins [5, 6], activation of MAPK8/JNK [5, 6], and inhibition of heat shock response [7, 8]. Triptolide has also been shown to influence epigenetic modulation of genes by interaction with histone methyltransferase and demethylase [9]. In spite of the wide therapeutic properties of triptolide, poor water solubility has limited its clinical use in the past. However, recently a water-soluble analog of triptolide–Minnelide has shown promising results in pancreatic cancer cell lines, human xenograft models, as well as in mouse models of pancreatic cancer [10]. Minnelide has been shown to reduce tumor burden in preclinical models of osteosarcoma [11]. Taken together with the anti-tumor properties of triptolide and the recent development of triptolide analogs to overcome its water solubility, triptolide has emerged as a promising anti-tumor agent.

In the present study, we evaluated the impact of genetic variations and gene expression profiles predictive of triptolide cytotoxicity using Epstein-Barr-virus transformed lymphoblastoid cell lines (LCLs) that are part of International HapMap project (www.hapMap.org) [12]. HapMap LCLs has been used as model to identify genetic markers associated with in vitro chemo-sensitivity to several drugs [13–16]. Genotype data is publically available allowing for genome-wide association analyses for biomarker identification. Our results validated some of the known genes/pathways as well as identified novel candidate genes/pathways of relevance to triptolide. We further validated the functional significance of CFLAR in pancreatic cell lines.

## Methods

### In vitro cytotoxicity assays

HapMap LCLs from subjects with European ancestry (CEU; n = 55 unrelated) were obtained from the Coriell Institute for Medical Research and were maintained as recommended. In vitro cytotoxicity was determined by treating LCLs with varying concentrations of triptolide (500, 50, 20, 10, 6.67,1.3 and 0 nM) for 48 hr followed by cell viability measurements using MTT (3-(4,5-dimethylthiazol-2-yl)-2,5-diphenyltetrazolium bromide assays (Life Technologies, USA) and Synergy 5 multi-plate reader. Cytotoxicity assays were performed in duplicates and cell lines were randomly chosen to repeat on different dates to rule out any experimental variation. Panc-1, a pancreatic cell line (ATCC, USA) was used for functional validation of the top gene identified in the GWAS. Panc-1 was cultured in DMEM medium supplemented with 2 mM glutamine and 10 % fetal bovine serum.

### Real-time quantitative PCR analysis

mRNA expression levels of CFLAR spliced isoforms were quantitated in LCLs and Panc-1 cell lines using CFLAR isoform specific oligonucleotide primers and 2^-ΔΔCT method as described in Additional file 1 and Fig. 2.

### Genotyping of panc-1 cell lines

Genomic DNA from Panc-1 cell line was genotyped for CFLAR SNP rs10190751 (A/G) using TaqMan SNP genotyping assay (Applied Biosystems, Foster City, CA, USA). Genomic DNAs from HapMap cell lines with known genotype (AA, AG and GG) were used as controls.

### Western blotting

Western plotting was performed using whole-cell lysates and CFLAR (Enzo lifesciences) or β-actin (Abcam) primary and mouse IgG secondary antibodies.

### siRNA mediated knockdown of CFLAR in cancer cell lines

Mission esiRNA were procured from Sigma Aldrich. esiRNA were synthesized through in vitro transcription of a 300–600 bp gene specific dsRNA, which is further digested in to complex pool of siRNA using RNAses. This digested pools of esiRNA are verified by DNA sequensing and gel electrophoresis to ensure identity and high specificity (Sigma Aldrich). To ensure high specificity and efficacy of the esiRNA, the algorithm DEQOR is utilized (Design and Quality Control of RNAi, available to the public via http://deqor.mpi-cbg.de/deqor_new/input.html). CFLAR specific 424 base pair sequence used in this study to create esiRNA pool for CFLAR knockdown is “TCCATCAGGTTGAAGAAGCACTTG ATACAGATGAGAAGGAGATGCTGCTCTTTTTGTGCCGGGATGTTGCTATAGATGTGGTTCCACCTAATGTCAGGGACCTTCTGGATATTTTACGGGAAAGAGGTAAGCTGTCTGTCGGGGACTTGGCTGAACTGCTCTACAGAGTGAGGCGATTTGACCTGCTCAAACGTATCTTGAAGATGGACAGAAAAGCTGTGGAGACCCACCTGCTCAGGAACCCTCACCTTGTTTCGGACTATAGAGTGCTGATGGCAGAGATTGGTGAGGATTTGGATAAATCTGATGTGTCCTCATTAATTTTCCTCATGAAGGATTACATGGGCCGAGGCAAGATAAGCAAGGAGAAGAGTTTCTTGGACCTTGTGGTTGAGTTGGAGAAACTAAATCTGGTTGCCCCAGA”.

Panc-1 cells were transfected with 200 nM CFLAR-esiRNA and negative siRNA using Lipofectamine® 2000 (Life technologies) as per manufacturer’s protocol. Twenty-four hours post-transfections cell were treated with varying concentrations of triptolide and cell viability was determined 48 hr post-treatment using MTT assays. mRNA levels of all three isoforms of CFLAR siRNA were quantitated 24 hr post-transfection to check for the knockdown.

### Transfection of CFLAR-Short and CFLAR-Long plasmid in Panc-1 cell line

Panc-1 cells were transfected with control and CFLAR expression plasmids (pEF6-V5, pEF-Flag A, pEF6-V5-CFLAR-S and pEF-Flag A-CFLAR-L) using Fugene HD reagent (Promega) followed by triptolide treatment and MTT assay as described above. Cell pellets were also collected for protein analysis.

### Statistical analysis

As these cell lines are part of several publically available genotyping databases, genotype data was retrieved on all cell lines from the HapMap project (release 23). For 29 samples, data was also retrieved from the 1000 genomes project (20101123 version). mRNA expression was retrieved for all of the individuals from a publically available source (http://www.sanger.ac.uk/research/areas/humangenetics/). IC_50_, concentration that kills 50 % of the cells, was calculated from a 4-parameter logistic model using the package drc v2.2.1 in R v2.14.0 [17].

### Association analysis of triptolide cytotoxicity with genetic variation

SNP genotype (n = 4098136) data was retrieved from the HapMap (release 23) for all 55 samples (29 female and 26 male). SNPs were filtered using various quality control criteria as, build changes, call rate, compliance with Hardy Weinberg and minimum allele frequency (MAF) as described in Additional file 1. In total 1978803 SNPs in 55 individuals passed quality control measures. For genotype data for individuals in the 1000 genomes only a MAF filter was used, dropping SNPs with MAF < 0.05. Several SNPs overlap between the 1000 genomes and HapMap data, data obtained from the 1000 genomes project was used preferentially over data obtained from the HapMap. Since not all of the samples used have been sequenced as part of the 1000 genomes project as of current, SNPs in the 1000 genomes project, but not in HapMap (release 23) were imputed for samples not included in the 1000 genomes project. BEAGLE v3.3.1 [18] was used to impute SNPs with the reference of the 1000 genomes as described in Additional file 1.

### Association analysis of triptolide cytotoxicity with gene expression variation

mRNA expression from the Illumina Sentrix Human-6 Expression BeadChip version 1 was normalized as per Stranger et al., using 47293 probes [19]. For this analysis, only the Caucasian samples were used and data was normalized using quantile normalization across replicates and median normalization across individuals. Original Illumina annotation was retrieved from ReMOAT [20]. Expression was not adjusted for gender (did not appear to affect association with phenotypes).

### Integrative analyses

With the wealth of data produced by current genomic technologies, collection of multiple types of genomic data on a set of samples is becoming commonplace. New methods explore a multifactor approach that combine different kinds of genomic data, sometimes referred to as “integrative genomics” or “genomic convergence”, in which a multistep procedure is used to identify potential key drivers of complex traits that integrate DNA variation and gene expression data. To integrate the genotype, expression and drug cytotoxicity data, we first identified markers associated with triptolide IC_50_ using a liberal significance threshold of 0.001. Next, we determined which expression probe sets were associated with these IC_50_ associated SNPs (*p*-values ≤ 10^−5^) (i.e., eQTLs). Finally, to determine whether the expression probe sets associated with these SNPs were also associated with triptolide IC_50_ values, we identified which expression probe sets were associated with IC_50_ with a *p*-values ≤ 0.0001). A similar integrative analysis approach has been used successfully to detect novel candidate genes [21]. The association between IC_50_ and SNP genotypes modeled as count of rare alleles (additive genetic model), or log_2_ normalized mRNA expression and cytotoxicity phenotypes, or SNPs was quantified using a spearman correlation coefficient, and *p*-value calculated for the null hypothesis of no association using an F-test.

## Results

### Genetic associations with triptolide IC_50_ in LCLs

**Fig. 1:**
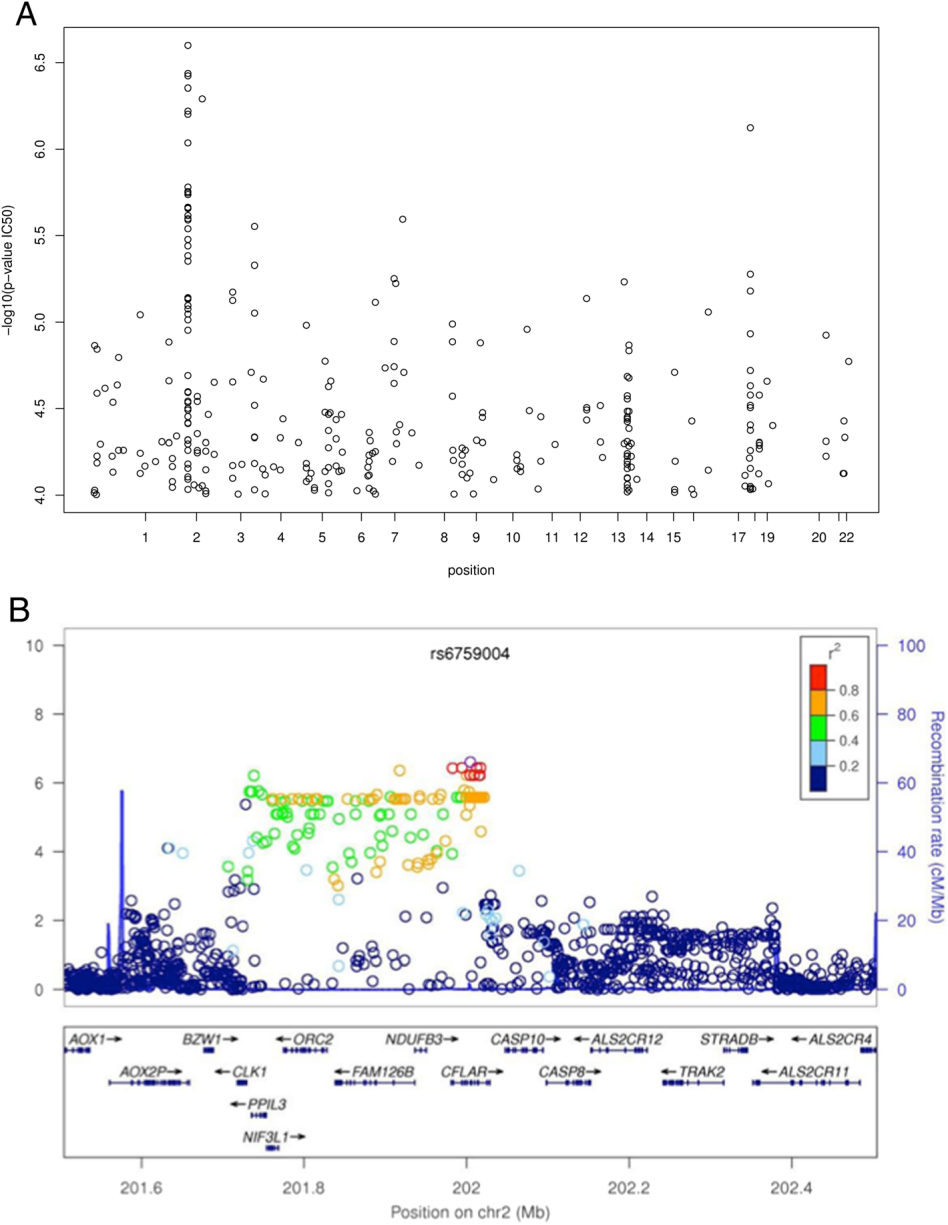
Genome-wide association study of SNPs with Triptolide cytotoxicity. **a** Manhattan plot showing association of SNPs with Triptolide IC_50_ (only SNPs with p <10^−4^ are included). **b** Genomic region on Chr 2 with strongest association with triptolide cytotoxicity. Y-axis represents -Log _10_ (P value) and X-axis presents chromosomal location

We evaluated 55 LCLs derived from unrelated subjects with Caucasian ancestry for cellular sensitivity to triptolide. Triptolide IC_50_ values ranged from 4 to 34 nM indicating wide inter-individual variation in chemo-sensitivity. GWAS analysis identified 140 SNPs in 11 genes that were associated with triptolide IC_50_ at p < 10^−5^ (488 SNPs at p < 10^−4^; Fig. 1a). Significant proportion of SNPs (110 of 140: 78.6 %) clustered in ~293 kb region on chromosome 2 (Table 1), which maps to multiple biologically interesting genes (Genes important for cell division and Cancer development) including *CFLAR, CLK1, FAM126B, NDUFB3, NIF3L1, ORC2* and *PPIL 3* (Table 2; Fig. 1b). Other genes with significant SNPs included TP53BP2 (chr 1), MTSU2 (chr 13), ZNF532 (chr18) and FNDC3B (chr 3). Ingenuity pathway analysis of these genes mapped them to 4 networks (Table 3). These networks are involved in Cellular Movement, Inflammatory Response, Cell-To-Cell Signaling and Interaction; Cell Death and Survival, Cellular Function and Maintenance, Molecular Transport; Cancer, Dermatological Diseases and Conditions, Developmental Disorder; Cellular Movement, Hematological System Development and Function, Immune Cell Trafficking and carbohydrate metabolism.

**Table 1:** List of top 140 SNPs (p <0.00001) from GWAS analysis that were predictive of triptolide cytotoxicity in HapMap LCLs

SNP	Position	Minor Allele	MAF	r.ic_50_	p value	Chr	Gene_function_list
rs6759004	202004676	C	0.15	−0.63	2.51E-07	2	CFLAR/intron
rs10931931	201994540	C	0.15	−0.62	3.66E-07	2	CFLAR/UTR-5
rs13413479	202012578	G	0.15	−0.62	3.66E-07	2	CFLAR/intron
rs73045343	202017408	G	0.15	−0.62	3.66E-07	2	CFLAR/intron
rs6712963	201982693	G	0.15	−0.62	3.78E-07	2	CFLAR/intron
rs10469755	201917938	T	0.17	−0.62	4.43E-07	2	FAM126B/intron
rs13059218	26910495	A	0.24	0.62	5.11E-07	3	
rs73045306	202000395	C	0.17	−0.61	6.01E-07	2	CFLAR/intron
rs6759216	202004710	G	0.17	−0.61	6.01E-07	2	CFLAR/intron
rs10203550	202009586	G	0.17	−0.61	6.01E-07	2	CFLAR/intron
rs13426823	202015677	G	0.17	−0.61	6.01E-07	2	CFLAR/intron
rs12105811	202016245	A	0.17	−0.61	6.01E-07	2	CFLAR/intron
rs73045341	202016550	G	0.17	−0.61	6.01E-07	2	CFLAR/intron
rs111315781	201738724	T	0.14	−0.61	6.27E-07	2	PPIL3/intron
rs73439953	56602702	T	0.33	−0.61	7.51E-07	18	ZNF532/intron
c2.201734921.b37p0	201734921	G	0.14	−0.61	9.19E-07	2	
rs55953858	201997292	G	0.21	−0.60	1.66E-06	2	CFLAR/intron
rs4035022	201744352	A	0.15	−0.59	1.76E-06	2	PPIL3/intron
rs3851973	201732878	G	0.14	−0.59	1.79E-06	2	PPIL3/UTR-5 and CLK1/UTR-3
rs111976464	201735042	C	0.14	−0.59	1.79E-06	2	PPIL3/UTR-5 and CLK1/UTR-3
rs11892119	201736112	C	0.14	−0.59	1.79E-06	2	PPIL3/UTR-3
rs7562391	201736166	C	0.14	−0.59	1.79E-06	2	PPIL3/missense
rs7560613	202003010	C	0.21	−0.60	1.83E-06	2	CFLAR/intron
rs6747253	201748415	A	0.15	−0.59	2.17E-06	2	PPIL3/intron
rs73043383	201968246	C	0.17	−0.59	2.19E-06	2	CFLAR/UTR-5
rs7591472	201889751	C	0.17	−0.59	2.20E-06	2	FAM126B/intron
rs10194347	201942480	A	0.17	−0.59	2.42E-06	2	NDUFB3/intron
rs28405687	201906661	T	0.23	−0.59	2.52E-06	2	FAM126B/intron
rs915650	37657526	A	0.38	−0.59	2.54E-06	8	
rs7583529	201988238	A	0.21	−0.59	2.57E-06	2	CFLAR/intron
rs6728771	201992407	A	0.21	−0.59	2.57E-06	2	CFLAR/intron
rs6706980	201993688	A	0.21	−0.59	2.57E-06	2	CFLAR/intron
rs58886660	201999283	A	0.21	−0.59	2.57E-06	2	CFLAR/intron
rs28636431	201999550	C	0.21	−0.59	2.57E-06	2	CFLAR/intron
rs7573256	202003170	G	0.21	−0.59	2.57E-06	2	CFLAR/intron
rs113350756	202003767	G	0.21	−0.59	2.57E-06	2	CFLAR/intron
**rs10190751**	**202006096**	**A**	**0.21**	**−0.59**	**2.57E-06**	**2**	**CFLAR/intron**
rs56160734	202006860	A	0.21	−0.59	2.57E-06	2	CFLAR/intron
rs4487072	202008298	T	0.21	−0.59	2.57E-06	2	CFLAR/intron
rs1981726	202008700	A	0.21	−0.59	2.57E-06	2	CFLAR/intron
rs7573529	202010626	T	0.21	−0.59	2.57E-06	2	CFLAR/intron
rs13387186	202012068	A	0.21	−0.59	2.57E-06	2	CFLAR/intron
rs13413075	202012363	C	0.21	−0.59	2.57E-06	2	CFLAR/intron
rs12104442	202013956	C	0.21	−0.59	2.57E-06	2	CFLAR/intron
rs12721505	202014238	G	0.21	−0.59	2.57E-06	2	CFLAR/intron
rs7571899	202015028	G	0.21	−0.59	2.57E-06	2	CFLAR/intron
rs7585993	202015114	T	0.21	−0.59	2.57E-06	2	CFLAR/intron
rs61289882	202015250	A	0.21	−0.59	2.57E-06	2	CFLAR/intron
rs68044560	202017080	T	0.21	−0.59	2.57E-06	2	CFLAR/intron
rs56863085	202017647	C	0.21	−0.59	2.57E-06	2	CFLAR/intron
rs719125	202017860	T	0.21	−0.59	2.57E-06	2	CFLAR/intron
rs56009967	202019094	C	0.21	−0.59	2.57E-06	2	CFLAR/intron
rs56161269	202019210	A	0.21	−0.59	2.57E-06	2	CFLAR/intron
rs2041766	202021495	T	0.21	−0.59	2.57E-06	2	CFLAR/intron
rs4482462	202021747	C	0.21	−0.59	2.57E-06	2	CFLAR/intron
rs75518195	65067404	G	0.36	0.58	2.80E-06	4	nearest gene (~150 kb down TECRL)
rs13384245	201760937	G	0.17	−0.58	2.89E-06	2	NIF3L1/intron
rs10204787	201762147	A	0.17	−0.58	2.89E-06	2	NIF3L1/intron
rs113388793	201771341	G	0.17	−0.58	2.89E-06	2	NIF3L1/UTR-3 and ORC2/UTR-3
rs2307358	201785657	T	0.17	−0.58	2.89E-06	2	ORC2/Intron
rs2307362	201785837	A	0.17	−0.58	2.89E-06	2	ORC2/cds-synon
rs4622700	201793113	T	0.17	−0.58	2.89E-06	2	ORC2/Intron
rs9288314	201794173	T	0.17	−0.58	2.89E-06	2	ORC2/Intron
rs10185727	201803711	G	0.17	−0.58	2.89E-06	2	ORC2/intron
rs13408964	201804972	T	0.17	−0.58	2.89E-06	2	ORC2/intron
rs16836160	201813830	G	0.17	−0.58	2.89E-06	2	ORC2/Intron
rs13429609	201816257	T	0.17	−0.58	2.89E-06	2	ORC2/Intron
rs13392371	201816421	A	0.17	−0.58	2.89E-06	2	ORC2/Intron
rs16836477	201853457	G	0.17	−0.58	2.89E-06	2	FAM126B/intron
rs6719992	201871978	G	0.17	−0.58	2.89E-06	2	FAM126B/intron
rs13405753	201881677	G	0.17	−0.58	2.89E-06	2	FAM126B/intron
rs10197103	201885293	T	0.17	−0.58	2.89E-06	2	FAM126B/intron
rs13384791	201911164	G	0.17	−0.58	2.89E-06	2	FAM126B/intron
rs13417843	201913429	G	0.17	−0.58	2.89E-06	2	FAM126B/intron
rs10207746	201916418	C	0.17	−0.58	2.89E-06	2	FAM126B/intron
rs73043345	201920060	G	0.17	−0.58	2.89E-06	2	FAM126B/intron
rs13389349	201920618	C	0.17	−0.58	2.89E-06	2	FAM126B/intron
rs6737556	201924078	T	0.17	−0.58	2.89E-06	2	FAM126B/intron
rs7590522	201924788	T	0.17	−0.58	2.89E-06	2	FAM126B/intron
rs10194168	201942437	T	0.17	−0.58	2.89E-06	2	NDUFB3/intron
rs10205971	201958201	G	0.17	−0.58	2.89E-06	2	NDUFB3/UTR-5 and CFLAR/UTR-5
rs2110728	201965678	A	0.17	−0.58	2.89E-06	2	CFLAR/UTR-5
rs13421776	201760902	G	0.19	−0.58	3.33E-06	2	NIF3L1/intron
rs4381763	201776988	G	0.19	−0.58	3.33E-06	2	ORC2/intron
rs10183261	201797676	A	0.19	−0.58	3.33E-06	2	ORC2/intron
rs10205561	201809704	T	0.19	−0.58	3.33E-06	2	ORC2/Intron
rs2307357	201824092	A	0.19	−0.58	3.33E-06	2	ORC2/Intron
rs3087357	201828365	G	0.19	−0.58	3.33E-06	2	ORC2/UTR-5
rs874358	201829602	G	0.19	−0.58	3.33E-06	2	ORC2/UTR-5
rs3900738	201863803	A	0.19	−0.58	3.33E-06	2	FAM126B/intron
rs16836568	201879948	G	0.19	−0.58	3.33E-06	2	FAM126B/intron
rs13416500	201771798	A	0.18	−0.58	3.63E-06	2	NIF3L1/UTR-3 and ORC2/UTR-3
rs13404596	201771801	T	0.19	−0.58	3.63E-06	2	NIF3L1/UTR-3 and ORC2/UTR-3
rs6757272	201728141	A	0.35	−0.58	4.14E-06	2	CLK1/intron
rs10184098	202004190	G	0.21	−0.57	4.45E-06	2	CFLAR/intron
rs28447044	64903606	T	0.46	0.57	4.69E-06	4	nearest gene (250 kb down TECRL)
rs11660954	56603803	G	0.26	−0.57	5.27E-06	18	ZNF532/intron
rs34044649	155655693	T	0.42	−0.57	5.59E-06	7	
c14.20107162.b37p0	20107162	C	0.24	0.73	5.84E-06	14	
rs2950160	4420188	G	0.31	−0.57	5.96E-06	8	CSMD1/intron
c18.56609415.b37p0	56609415	T	0.24	−0.57	6.60E-06	18	
rs9854411	171921569	C	0.16	−0.57	6.70E-06	3	FNDC3B/intron
rs13412214	201774182	T	0.23	−0.56	7.21E-06	2	NIF3L1/UTR-3 and ORC2/UTR-3
rs13412430	201774355	T	0.23	−0.56	7.21E-06	2	NIF3L1/UTR-3 and ORC2/UTR-3
rs10172647	201778950	A	0.23	−0.56	7.29E-06	2	ORC2/intron
rs3125719	29990966	T	0.18	−0.56	7.30E-06	13	MTUS2/intron
c2.201899549.b37p0	201899549	C	0.17	−0.56	7.35E-06	2	
rs6445044	171920569	G	0.16	−0.56	7.48E-06	3	FNDC3B/intron
rs6414541	171920637	C	0.16	−0.56	7.48E-06	3	FNDC3B/intron
rs3923825	171922127	T	0.16	−0.56	7.48E-06	3	FNDC3B/intron
rs3924140	171926314	C	0.16	−0.56	7.48E-06	3	FNDC3B/intron
rs4610256	171928021	C	0.16	−0.56	7.48E-06	3	FNDC3B/intron
rs6445045	171928400	C	0.16	−0.56	7.48E-06	3	FNDC3B/intron
rs9867872	171928985	C	0.16	−0.56	7.48E-06	3	FNDC3B/intron
rs9868872	171929530	G	0.16	−0.56	7.48E-06	3	FNDC3B/intron
rs4380442	171930381	A	0.16	−0.56	7.48E-06	3	FNDC3B/intron
rs6763764	171931389	C	0.16	−0.56	7.48E-06	3	FNDC3B/intron
rs6803181	171932256	C	0.16	−0.56	7.48E-06	3	FNDC3B/intron
rs6780871	171932606	G	0.16	−0.56	7.48E-06	3	FNDC3B/intron
rs6806070	171932935	C	0.16	−0.56	7.48E-06	3	FNDC3B/intron
rs7619745	171933571	A	0.16	−0.56	7.48E-06	3	FNDC3B/intron
rs2016325	66323500	T	0.35	0.56	7.68E-06	7	
rs6435066	201766023	G	0.23	−0.56	8.00E-06	2	NIF3L1/intron
rs7917	201768238	T	0.23	−0.56	8.00E-06	2	NIF3L1/missense
rs11894842	201773545	C	0.23	−0.56	8.00E-06	2	NIF3L1/UTR-3 and ORC2/UTR-3
rs10167387	201808618	C	0.23	−0.56	8.00E-06	2	ORC2/Intron
rs68133847	201812251	G	0.23	−0.56	8.00E-06	2	ORC2/Intron
rs11892372	201819483	A	0.23	−0.56	8.00E-06	2	ORC2/Intron
rs3901120	201863530	C	0.23	−0.56	8.00E-06	2	FAM126B/intron
rs16836591	201894943	T	0.23	−0.56	8.00E-06	2	FAM126B/intron
rs10185136	201904301	T	0.23	−0.56	8.00E-06	2	FAM126B/intron
rs10197833	201904466	C	0.23	−0.56	8.00E-06	2	FAM126B/intron
rs16842071	201931730	C	0.23	−0.56	8.00E-06	2	FAM126B/intron
rs13410282	201971024	A	0.23	−0.56	8.00E-06	2	CFLAR/UTR-5
rs16836710	201999845	T	0.19	−0.57	8.39E-06	2	CFLAR/intron
rs61457372	70359774	C	0.35	0.56	8.73E-06	17	
rs6841405	65067406	G	0.4	0.56	8.86E-06	4	nearest gene (~150 kb down TECRL)
rs7597409	201893188	T	0.23	−0.56	8.97E-06	2	FAM126B/intron
rs12723025	223977519	T	0.073	0.56	9.06E-06	1	TP53BP2/intron
rs6754564	201779932	C	0.16	−0.56	9.69E-06	2	ORC2/Intron

**Table 2:** Summary of genes with SNPs significantly associated (p < 0.00001) with triptolide cytotoxicty in HapMAP LCLs

Gene Symbol	Gene full name	Gene summary (Ref gene)	# of SNPs at p < 0.00001
CFLAR	Caspase 8 and FADD-like apoptosis regulator	The protein encoded by this gene is a regulator of apoptosis and is structurally similar to caspase-8. However, the encoded protein lacks caspase activity and appears to be itself cleaved into two peptides by caspase-8. Several transcript variants encoding different isoforms have been found for this gene, and partial evidence for several more variants exists	45
FAM126B	Family With Sequence Similarity 126, Member B		22
PPIL3	Peptidylprolyl isomerase 9cyclophilin)-like 3	This gene encodes a member of the cyclophilin family. Cyclophilins catalyze the cis-trans isomerization of peptidylprolyl imide bonds in oligopeptides. They have been proposed to act either as catalysts or as molecular chaperones in protein-folding events. Alternative splicing results in multiple transcript variants. PPIases accelerate the folding of proteins. It catalyzes the cis-trans isomerization of proline imidic peptide bonds in oligopeptides.	7
ZNF532			2
NDUFB3	NADH dehydrogenase (ubiquinone0 1beta subcomplex,3, 12kD	This gene encodes an accessory subunit of the mitochondrial membrane respiratory chain NADH dehydrogenase (Complex I)which is the first enzyme in the electron transport chain of mitochondria. This protein localizes to the inner membrane of the mitochondrion as a single-pass membrane protein. Mutations in this gene contribute to mitochondrial complex 1 deficiency	3
NIF3L1	NIF3 NGG1 interacting factor 3-like 1(S. cerevisiae)	Functions as transcriptional correpressor	11
ORC2	Origin recognition complex subunit 2	The origin recognition complex (ORC) is a highly conserved six subunits protein complex essential for the initiation of the DNA replication in eukaryotic cells. Studies in yeast demonstrated that ORC binds specifically to origins of replication and serves as a platform for the assembly of additional initiation factors such as Cdc6 and Mcm proteins. The protein encoded by this gene is a subunit of the ORC complex. This protein forms a core complex with ORC3,−4, and−5. It also interacts with CDC45 and MCM10, which are proteins known to be important for the initiation of DNA replication.	20
CLK1	CDC-like kinae 1	This gene encodes a member of the CDC2-like (or LAMMER) family of dual specificity protein kinases. In the nucleus, the encoded protein phosphorylates serine/arginine-rich proteins involved in pre-mRNA processing, releasing them into the nucleoplasm. The choice of splice sites during pre-mRNA processing may be regulated by the concentration of transacting factors, including serine/arginine rich proteins. Therefore, the encoded protein may play an indirect role in governing splice site selection. Phosphorylates: SRSF1, SRSF3 and PTPN1. Regulates the alternative splicing of tissue factor (F3) pre-mRNA in endothelial cells and adenovirus E1A pre-mRNA	1
CSMD1	CUB and Sushi multiple domains 1		1
FNDC3B	fibronectin type III domain containing 3B		15
MTUS2	microtubule associated tumor suppressor candidate 2	Binds microtubules. Together with MAPRE1 may target the microtubule depolymerase KIF2C to the plus-end of microtubules. May regulate the dynamics of microtubules at their growing distal tip	1
TP53BP2	tumor protein p53 binding protein, 2	This gene encodes a member of the ASPP (apoptosis-stimulating protein of p53) family of p53 interacting proteins. The protein contains four ankyrin repeats and an SH3 domain involved in protein-protein interactions. It is localized to the perinuclear region of the cytoplasm, and regulates apoptosis and cell growth through interactions with other regulatory molecules including members of the p53 family. It plays central role in regulation of apoptosis and cell growth via its interactions. Regulates TP53 by enhancing the DNA binding and transactivation function of TP53 on the promoters of proapoptotic genes in vivo. Inhibits the ability of APPBP1 to conjugate NEDD8 to CUL1, and thereby decreases APPBP1 ability to induce apoptosis. Impedes cell cycle progression at G2/M. Its apoptosis-stimulating activity is inhibited by its interaction with DDX42	1

**Table 3:** Ingenuity pathway analysis tool mapped the genes identified in gene expression vs. cytotoxicity analysis to 5 networks

ID	Molecules in Network	Score	Focus Molecules	Top Functions
1	arginase,BCL6,BCR (complex),CXCL9,ERK1/2,Fc gamma receptor,Fcgr3,GCNT2,Gm-csf,GOT,IFN Beta,IFN type 1,Iga,Ige,IgG1,IgG,IgG2a,IgG2b,Igm,IL13,IL19,IL-2R,IL12 (complex),IL12 (family),IL12RB2,IL2RA,Immunoglobulin,Interferon alpha,JAK1,myosin-light-chain kinase,PRF1,RAB27A,SLC12A6,TH2 Cytokine,THBD	26	12	Cellular Movement, Inflammatory Response, Cell-To-Cell Signaling and Interaction
2	26 s Proteasome,ADIRF,beta-estradiol,C16orf59,CLYBL,DNAH17,DPYSL3,FAM111A, FAM134C,FAS,FEZ1,FSH,GEMIN8,GPR107,Gsk3,GTPBP6,HAP1,HIST3H2BB,HS1BP3, KIAA0922,KRT83,LHFPL2,LOC391322,MAGEA8,MYEOV,PXMP4,RNF212,TMEM164, TMEM258,TMEM150C,UBC,UBIAD1,ZNF20,ZNF691,ZNF75D	21	10	Cell Death and Survival, Cellular Function and Maintenance, Molecular Transport
3	ABCB11,AFAP1L2,caspase,CHRNE,Ck2,CRTAC1,EDARADD,ERK,Hdac,Histone h3,Histone h4,HOXB6,Hsp90,Ifn,IFN alpha/beta,IFNG,IKK (complex),IL1,Insulin,Jnk,JUN,NFkB (complex),p85 (pik3r),Pka,PKP1,Pro-inflammatory Cytokine,Rac,Ras,Ras homolog,RNA polymerase II,Rxr,STAT,TCR,TSH,Vegf	18	9	Cancer, Dermatological Diseases and Conditions, Developmental Disorder
4	Akt,Ap1,calpain,CCND1,CD3,CDK20,Cg,CLDN11,Collagen type I,Collagen(s),Creb,CSF1,Cyclin A,Cyclin E,DTX1,Fcer1,GPC3,Hsp27,Ifn gamma,Integrin,LDL,MAP2K1/2,Mapk,MAPKAPK2,Mek,P38 MAPK,Pdgf (complex),PDGF BB,PI3K (complex),PLC gamma,Rb,Rock,STAT5a/b,Tgf beta,Tnf (family)	14	7	Cellular Movement, Hematological System Development and Function, Immune Cell Trafficking
5	D-xylose 1-dehydrogenase (NADP),DHDH,trans-1,2-dihydrobenzene-1,2-diol dehydrogenase	2	1	Carbohydrate Metabolism

CFLAR (Caspase 8 and FADD like apoptosis regulator) with maximum number of most significant SNPs (n = 41; due to high LD as shown in Additional file 2: Figure S1) codes for protein c-FLIP. c-FLIP regulates apoptosis and is structurally similar to caspase-8, however, lacks the caspase activity. It has been implicated as a crucial link between cell survival and cell death pathways in mammalian cells.

### Gene expression associations with triptolide IC_50_ in LCLs

Genome-wide gene expression analysis identified 14 probes that were associated with triptolide IC_50_ (p < 0.0001, Additional file 3: Figure S2 and Table 4). Some of the biologically interesting genes with expression levels associated with triptolide sensitivity included: JAK1 (Janus kinase 1), DTX1 (Deltex Homolog 1; positive regulator of Notch signaling pathway), AGL (Amylo-Alpha-1, 6-Glucosidase, 4-Alpha-Glucanotransferase involved in glycogen degradation), and MUC15 (Mucin 15, Cell Surface Associated). Pathway analysis using Ingenunity pathway analysis tool mapped these genes to JAK/STAT, IL, iNOS, EGFsignalining pathways (Additional file 4: Figure S3).

**Table 4:** Top probe and genes with expression levels in LCLs associated with Triptolide LC_50_

Probe	chromosome	start	Stop	gene	Spearman correlation	P-value
hmm21260-S	12	39215842	39215878	hmm21260	−0.563	7.62E-06
GI_21389558-S	12	128035309	128035358	FLJ31978	0.554	1.16E-05
Hs.390856-S	1	111024040	111024089	Hs.390856	−0.553	1.18E-05
Hs.469348-S	2	96793632	96793677	Hs.469348	−0.531	3.04E-05
GI_37547236-S	2	109782098	109782147	LOC376934	−0.53	3.20E-05
Hs.128030-S	5	55803569	55803618	Hs.128030	−0.522	4.33E-05
GI_37538743-S	7	99411521	99411570	LOC377544	−0.518	5.18E-05
GI_40255097-S	11	26541312	26541361	MUC15	−0.514	5.91E-05
GI_42660336-S	14	89994713	89994762	LOC400238	−0.514	5.97E-05
GI_41352717-S	12	112017575	112017602	DTX1	0.509	7.33E-05
hmm28068-S	2	85500058	85500086	hmm28068	−0.509	7.37E-05
GI_4557282-I	1	100099399	100099440	AGL; GDE	−0.507	7.75E-05
GI_4504802-S	1	65073667	65073716	JAK1; JAK1A	0.505	8.57E-05
hmm35855-S	9	116734605	116734654	hmm35855	−0.503	9.02E-05

### Integrated SNP-mRNA association analysis with IC_50_

We performed integrated analysis between SNPs-mRNA expression-IC_50_; we selected top SNPs that were significantly (p < 0.001) associated with cytotoxicity, and top gene expression signatures significantly (p < 0.001) associated with cytotoxicity for association with each other i.e. SNP vs. gene expression. This analysis basically identified eQTLs associated with triptolide IC_50_ and at p <0.0001 we identified 648 unique SNP-mRNA pairs that were associated with triptolide cytotoxicity however these SNP-mRNA pairs mapped to only 28 genes (Table 5), indicating association of multiple SNPs with one gene (which might be due to LD between SNPs). Some of the biologically interesting SNP-mRNA pairs included: association of multiple SNPs in chromosome 16 spanning genes CHST5, TMEM231, GABARAPL2 and ADAT1 with expression levels of AGL; SNPs in ASXL3 with ASCL4 expression; CAMTA1 SNPs and CRYGS expression; TIAM1 SNPs with DTX1 expression; SNPs in GPATCH1, and multiple SNPs on chr 5 and 14 with JAK1 expression. Multiple SNPs within CFLAR were associated with expression levels of MTVR1, PIP5K1B and C9orf19/GLRIP2.

**Table 5:** Summary of 3 way integrated analysis of Gene-Expression-SNP and triptolide cytotoxicity (p < 0.0001)

Gene expression (Gene symbol)	Gene description	Association with total # of SNPs (p < 0.0001)	Distribution of SNPs (Chr: number of SNPs)	Distribution of SNPs on gene (Bold indicates Maximum number of SNPs on Gene)
AGL (Chr1)	Amylo-Alpha-1, 6-Glucosidase, 4-Alpha-Glucanotransferase	27	Chr2:1 / Chr4:2 / Ch6:2 / Chr16:22	**CHST5**/ADAT1/GABARAPL2/LRP2/SCGN/TMEM231
ASCL4 (Chr12)	Achaete-Scute Complex-Like 4	24	Chr6:5 / Chr7:1 / Ch9:1 / Chr16:2 / Chr17:3 / Chr18:12	MPG/**ASXL3**/ATP6V0A1/EEPD1/MPG/NAGLU
C21orf96/RUNX-IT1 (Chr21)	RUNX1 Intronic Transcript 1 (Non-Protein Coding)	3	Chr5:1 / Chr9:1 / Chr13:1	PDZD2
C9orf19/GLIPR2 (Chr9)	Glioma Pathogenesis-Related Protein 2	20	Chr1:2 / Chr2:6 / Chr5, 7, 11 and 14 : 1 each / Chr8:3 / Chr12:2 / Chr13:3	ARRB1 / CAPN2 / **CFLAR/** TP53BP2
CRYGS (Chr3)	crystallin, gamma S	41	Chr1:30 Chr2 and Chr6: 3 each Chr5:2 Chr8, 9 and 14: 1 each	CAMTA1
CTSK (Chr1)	Cathepsin X	19	Chr1,9 and 16:1 each/ Chr5:6/ Chr7, 10 and 17: 2 each/ Chr14: 4 each	CRISPLD2/FHAD1/IDE/PTPRD/**RBFOX3/SNX6**/SP4
DTX1 (Chr12)	deltex homolog 1 (Drosophila)	31	Chr 6:5 / Chr 9:1 / Chr 14:2 / Chr 21:23	**TIAM1**
FOXQ1 (Chr6)	Forkhead Box Protein Q1	21	Chr 1:6 / Chr 3:10 / Chr 5 and 6:1 each / Chr 16:3	FLYWCH1/**GNG4**/PARK2
GABARAPL1 (Chr12)	GABA(A) Receptor-Associated Protein-Like 1	7	Chr 2:5 / Chr 6 and 14:1 each	STRN3
JAK1 (Chr1)	Janus Kinase 1	44	Chr 1:8 / Chr 2, 3, 6 and 10:2 each/ Chr 4, 12 and 13:1 each / Chr 5:12 / Chr 8:3 / Chr 14:10	**GPATCH2**/ SLC6A15/ TFDP2
MAPKAPK2 (Chr1)	Mitogen-Activated Protein Kinase-Activated Protein Kinase 2	3	Chr11:2 / Chr 5:1	**ARRB1/ OPCML**
MGC16186 (Chr10)	DPY30 Domain Containing 2	10	Chr 4:4 / Chr 7, 9 and 22:1 each / Chr 10:3	**MANBA/ MYO18B**
MGC16385 (Chr16)	CENPB DNA-Binding Domains Containing 1	18	Chr 4:4 / Chr 5:2 / Chr 17 and 22:1 each / Chr 14:10	AGXT2L1/ GIP / **ITPK1**/PDE6A
MTND4 (mitochondrial)	Mitochondrially Encoded NADH Dehydrogenase	16	Chr 1:8 / Chr 4, 6, 8, 9 and 14:1 each / Chr 10:3	GNG4/ **GPATCH2/ SH2D4B**
MTND5 (mitochondrial)	Mitochondrially Encoded NADH Dehydrogenase 5	10	Chr 9 and 10:1 / Chr 14:8	**PTGDR** / SH2D4B
MTVR1 (Chr11)	Mammary Tumor Virus Receptor Homolog 1	28	Chr 2:23 / Chr 6 and 10:1 each / Chr 7:3	**NIF3L1/ C7orf42/ CFLAR/ FAM126B/NIF3L1/ORC2**
MUC15 (Chr11)	Mucin 15, Cell Surface Associated	16	Chr 1:8 / Chr 2:2 / Chr 9, 15 and 21:1 each / Chr 11:3	**CAPN2/CGNL1/TP53BP2**
MXD4 (Chr4)	MAX dimerization protein 4	13	Chr 2:3 / Chr 4, 5, 10 and 17:1 each / Chr 9:2 / Chr 16:4	**MPG/NPRL3/CCDC46/KANK1/USP34**
NADSYN1 (Chr11)	NAD Synthetase 1	38	Chr 2:32 / Chr 13:1 / Chr 16:5	**ERBB4/ FHAD1/ GPATCH2**
PIP5K1B (Chr9)	Phosphatidylinositol 4-Phosphate 5-Kinase Type-1 Beta	75	Chr 1:2 / Chr 2: 65 / Chr 6 and 14:1 each / Chr 7:4 / Chr 13:2	**NIF3L1/ ABCC4/ CFLAR/ FAM126B/ GPATCH2/NDUFB3/NIF3L1/ORC2/PPIL3/SMYD3/TSGA14**
PODLX2 (Chr3)	podocalyxin-like 2	44	Chr 3 and 20:1 each / Chr 11:39 / Chr 11:3	**LDLRAD3**
ROPN1 (Chr3)	Rhophilin Associated Tail Protein 1	7	Chr 1:2 / Chr 3:3 / Chr 4 and 13:1	
SLC35D2 (Chr9)	Solute Carrier Family 35 Member D2	18	Chr 4 and 10:3 each / Chr 5:10 / Chr 13 and 22:1 each	**ABLIM2/ MANBA/ TTLL1**
TDE1 (Chr20)	Tumor Differentially Expressed 1	39	Chr 1:12 / Chr 2, 10and 14:1 each / Chr 3:6 / Chr 5:9 / Chr 8:7 / Cht 15:2	**FHAD1/PPCS/ SMYD3/ZMYND12**
TWSG1 (Chr18)	Twisted Gastrulation Protein Homolog 1	8	Chr 2:5 / Chr 6:3	**CLK1/ FAM126B/**
VIAAT (Chr20)	Solute Carrier Family 32 Member 1	55	Chr 6 and 10:4 each / Chr 12:1 / Chr 13: 3 / Chr 14:41 / Chr 16:2	**SNX6/ STRN3/ RSU1**
VPS39 (Chr15)	Vacuolar Protein Sorting 39	14	Chr 2, 3 and 5: 3 each / Chr 4, 11, 13, 14 and 22:1 each	**ACTN1/ ARRB1/ GRM7/ SEZ6L**
	Total	648		

### Functional significance of CFLAR splicing SNP

**Fig. 2:**
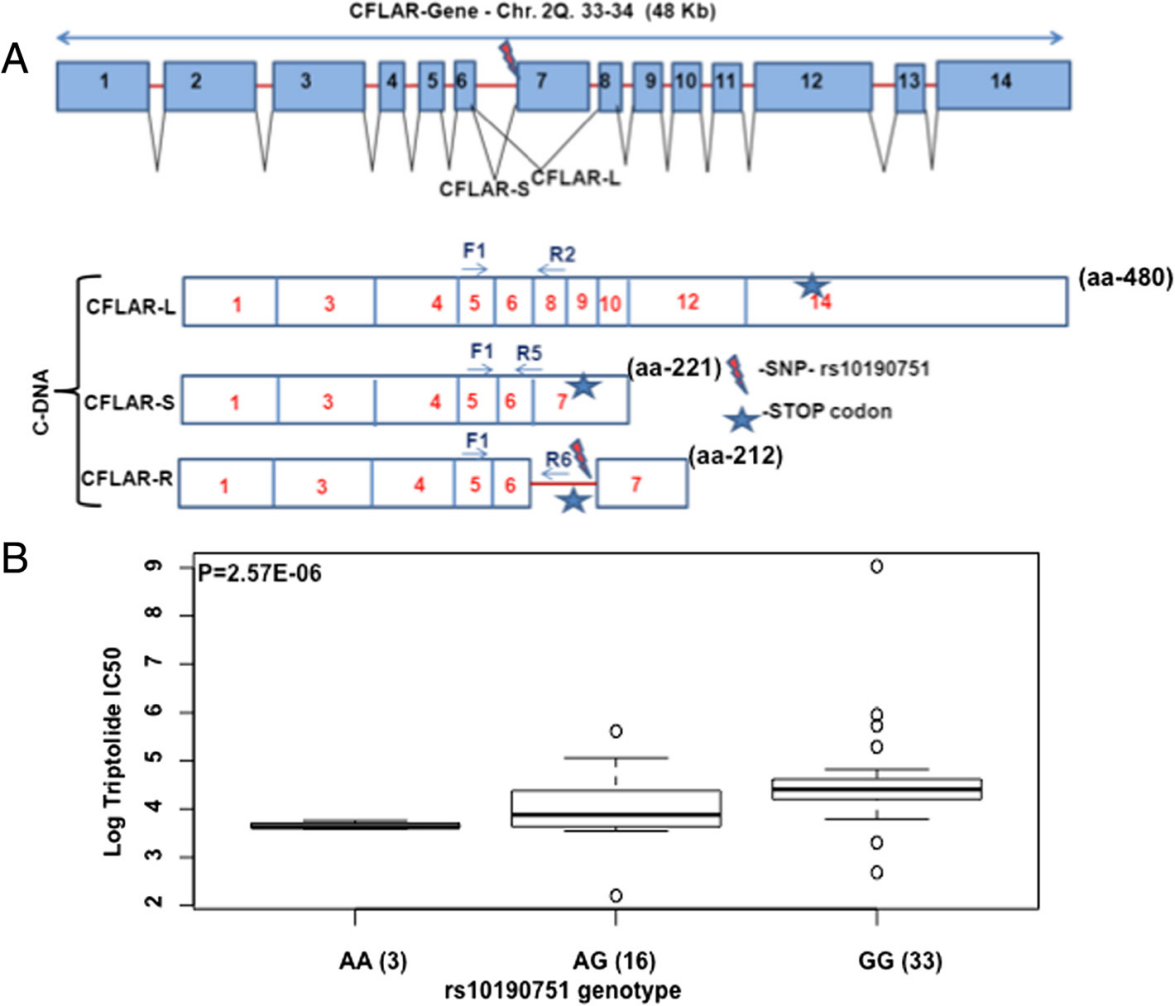
Schematic representation of CFLAR gene and its splice variants. **a** The schematic of CFLAR gene shows all the exons and the location of rs10190751 SNP at the 3’splice site of intron 6. Presence or absence of this SNP regulates production of CFLAR-short form (CFLAR-S). Difference splice variants of CFLAR are also shown along with the isoform specific primer pair’s used in this study. **b** Box plot showing association of rs10190751 Splicing SNP with Triptolide LC50 in HapMap LCLs. Y-axis is Log 2 Triptolide IC_50_ and X-axis represents rs10190751 genotype. Box Plots show medians as a line between boxes representing the first and third quartiles; the whiskers represent the range after excluding outliers. The outliers are defined as data points that fall outside of the first and third quartiles by more than 1.5-times the interquartile range. Circles falling outside the whiskers represent outliers

**Fig. 3:**
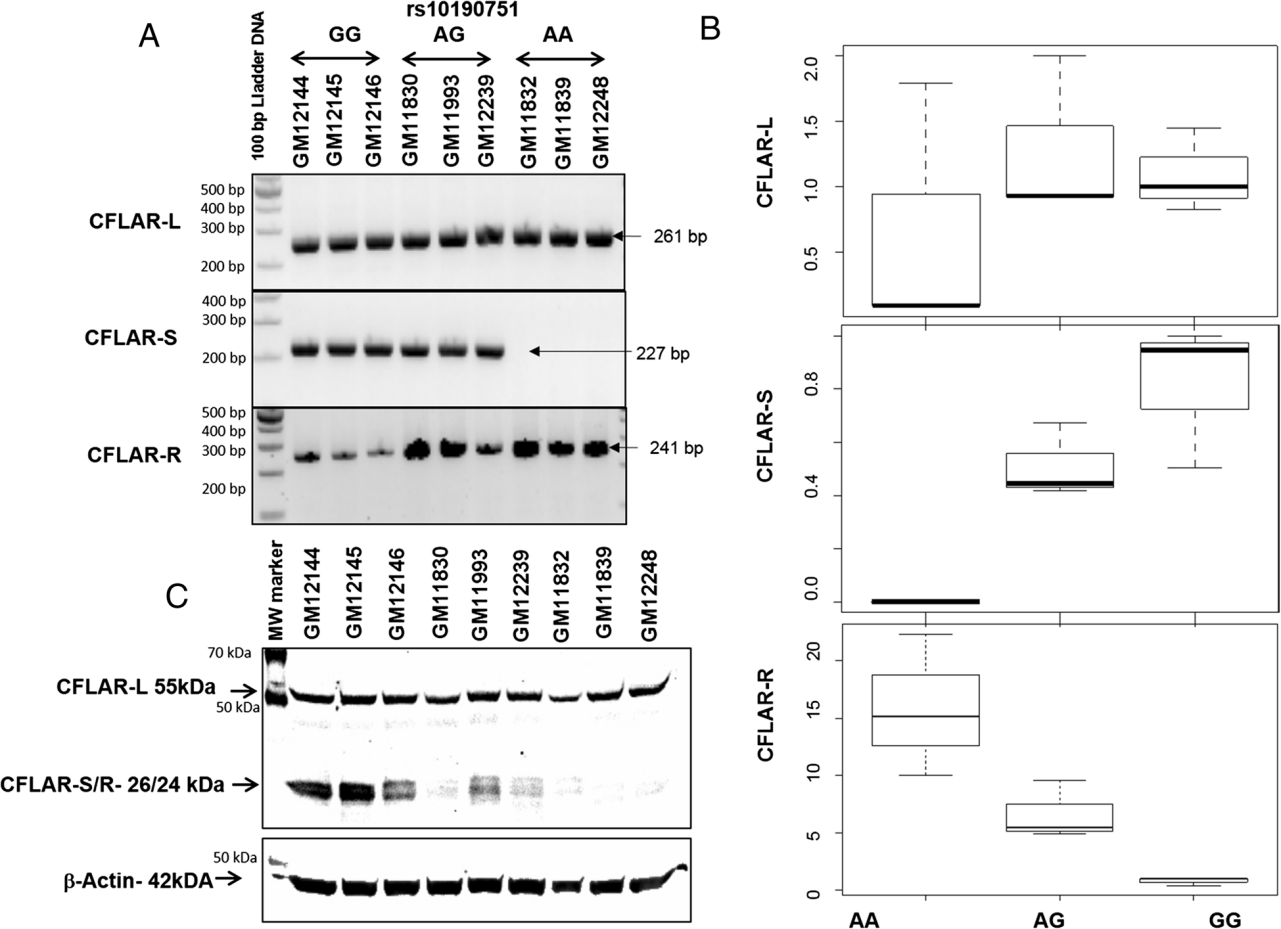
Correlation of rs10190751 genotype with CFLAR splice variants. **a** Isoform specific amplification of CFLAR splice-variants (CFLAR-L, CFLAR-S and CFLAR-R) in LCLs with AA, GG and AG genotype for rs10190751. **b** Real-time-mRNA quantification showing relative levels of CFLAR-L, CFLAR-S and CFLAR-R isoforms in different genotype groups. Box plots details are same as in figure 2B. **c** Western blotting showing protein levels of CFLAR-L and CFLAR-S/R forms in HapMap LCLs with AA, AG or GG genotype for rs10190751

We selected CFLAR for functional validation in our study on the basis of following observations; i) higher number of SNPs in CFLAR were associated with the triptolide cytotoxicity (Tables 1 and 2); ii) CFLAR. SNP-mRNA pairs associated with triptolide cytotoxicity included three CFLAR mRNA probes additionally multiple SNPs within CFLAR were associated with each 3 mRNA probes. Majority of the significantly associated SNPs within CFLAR gene were intronic and occurred in high LD (Additional file 2: Figure S1). However, one CFLAR SNP, rs10190751 G > A, was present at the splice junction of exon 7 (Fig. 2a) and was significantly associated with triptolide cytotoxicity (Fig. 2b). We screened HapMap cell lines with AA, AG and GG genotype (3 cell lines in each genotype group) for long (CFLAR-L), short (CFLAR-S) and raji (CFLAR-R) forms of CFLAR splice variants. CFLAR-L form was present in all cell lines irrespective of the genotype whereas CFLAR-S form was only expressed in cell lines with at least one G allele (Fig. 3a). Real-time quantification of CFLAR splice variants showed significant association of AA genotype with low levels of CFLAR-L; complete absence of CFLAR-S and higher levels of CFLAR-R form (Fig. 3). Western blot analysis confirmed the association of C-FLIP protein isoform levels corresponding to rs10190751 genotype and CFLAR mRNA isoforms (Fig. 3c).

### SiRNA mediated functional studies on CFLAR

**Fig. 4:**
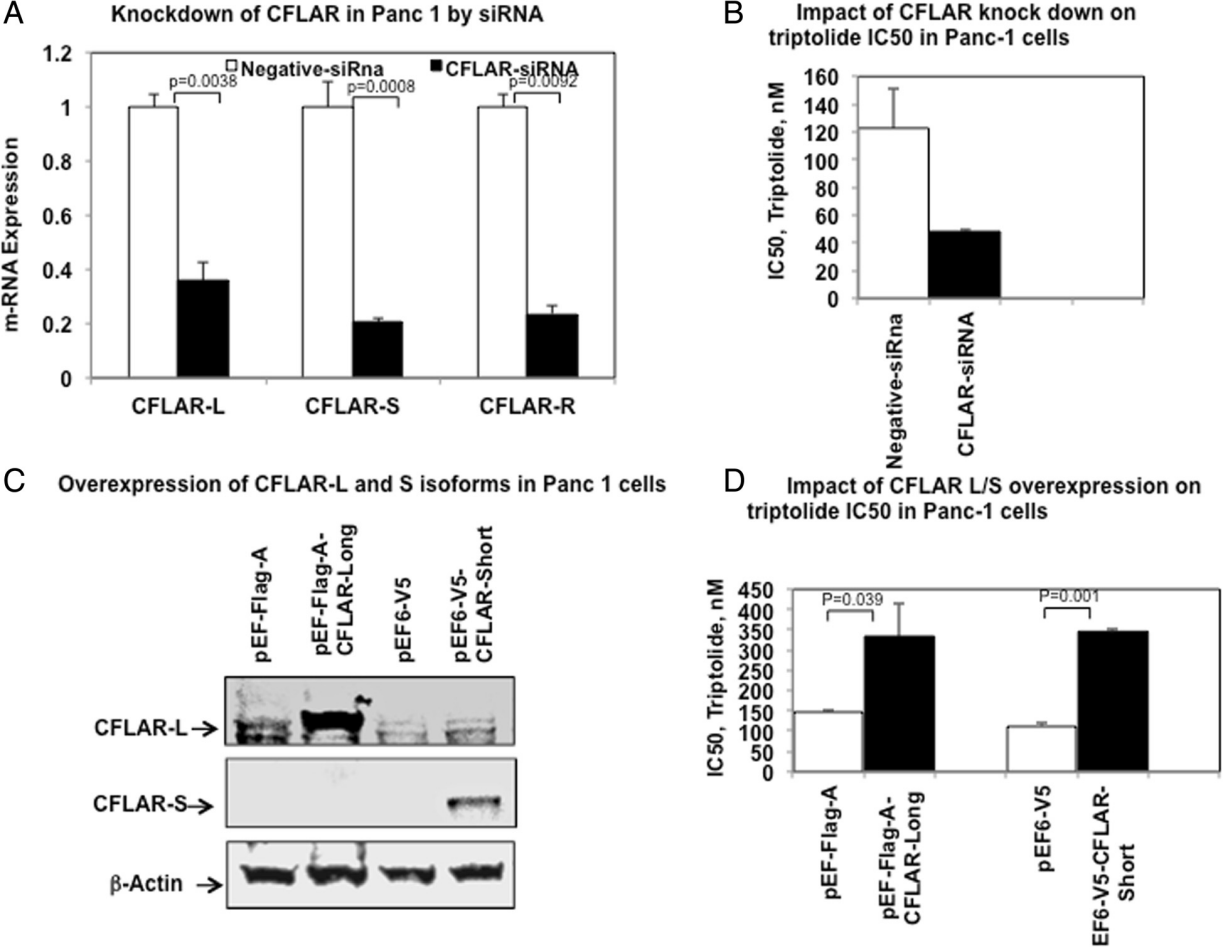
Impact of siRNA mediated knockdown or overexpression of CFLAR isoforms on Triptolide sensitivity in Panc-1 cancer cell lines. **a** Impact of siRNA medicated knockdown of CFLAR on mRNA expression levels of CFLAR-L, CFLAR-S and CFLAR-R isoforms **b** Impact of siRNA medicated knockdown of CFLAR on cellular cytotoxicity to Triptolide. **c** Western blot showing over expression of CFLAR-L and CLFAR-S isoforms as compared to cells transfected to control plasmids. **d** Impact of overexpression of CFLAR-L and CFLAR-S isoforms in cellular sensitivity to Triptolide

We selected Panc-1 for further investigation for impact of siRNA mediated transient knockdown of CFLAR on cellular sensitivity to triptolide. We selected Panc-1 for further investigation for impact of siRNA mediated transient knockdown of CFLAR on cellular sensitivity to triptolide. Panc-1 was selected based on the literature evidence of efficient use of triptolide in pancreatic cancer at pre-clinical and clinical level [Ref] as well as CFLAR being reported as a therapeutic target for triptolide in Pancreatic cancer [ref]. Genotype of Panc-1 for rs10190751, was identified as GG, therefore all isoform of CFLAR expressed in this cell line and it makes this cell line a perfect model to do functional validation for different isoform. In a pancreatic cancer cell line, Panc-1, siRNA mediated knockdown resulted in significant reduction in the CFLAR-L, CFLAR-S and CFLAR-R isoforms and significant increase in sensitivity to, triptolide (Fig. 4a and b).

### Over expression of CFLAR-long and short isoform

Since Panc-1 demonstrated change in chemo-sensitivity in siRNA mediated knockdown of CFLAR, we further overexpressed CFLAR-L and CFLAR-S forms in Panc-1 cell lines. Transient transfection of most abundant isoforms of CFLAR was done in Panc-1 cell line using pEFA-CFLAR-L and pEF6-V5-CFLAR-L plasmid for Long and Short form of CFLAR respectively. Compared to cells transfected with control plasmids the level of CFLAR protein isoforms were significantly increased (Fig. 4c). Over-expression CFLAR-L or CFLAR-S isoforms resulted in significant decrease in sensitivity for triptolide, (Fig. 4d).

## Discussion

Triptolide is a diterpenoid triepoxide and has been used in traditional Chinese medicine for years. Poor water solubility and toxicity has limited its use in clinics, however recent advances focused on developing triptolide derivatives with better solubility such as MC002 [22], omtritolide [23], minnelide [10] etc. are showing promising advancements especially in pancreatic cancer. The anticancer activity of triptolide has been associated with its ability to inhibit various pro-proliferative or anti-apoptotic factors thereby inducing apoptosis [16]. Triptolide has been implicated in activation of both intrinsic and extrinsic apoptotic pathways by inducing caspase-8, −9 and 3 as well as by inducing cleavage of PARP [24, 25]. Given the fact that triptolide has a promising potential as a therapeutic agent, we designed this study to identify the genomic markers associated with triptolide cytotoxicity using LCLs from International HapMap project.

Our results identified a QTL on chromosome 2 consisting of several SNPs with significant association with triptolide IC_50_. This region on chromosome 2 included biologically interesting genes such as CLK1, PPL3, NIF3L1, CFLAR, NDUFB3, CASP10, CASP8 etc. (Tables 1 and 2) with important roles in apoptosis and cell cycle regulation pathways. Of particular interest was significant over-representation the top most significant SNPs in CFLAR gene (Caspase 8 and FADD like apoptosis regulator). CFLAR gene encodes for protein c-FLIP, best-known for its anti-apoptotic regulatory role by inhibiting TNF-alpha, FAS-L and TRAIL induced apoptosis [26]. Although most of the SNPs in CFLAR were intronic, one splicing SNP, rs10190751 (3’ splice site of intron 6) was of particular interest [27]. CFLAR-protein, c-FILP exists in several isoforms due to alternate splicing, the most studied forms include long (C-FLIP-L) and short (C-FLIP-S) isoforms of 55 kD and 26 kD, respectively. *CFLAR* gene has 14 exons and inclusion or exclusion of intron 6 or exon 7 regulates the expression of long, or short or raji forms. CFLAR long form (CFLAR-L) skips exon 7 and is expressed as a full-length protein of 480 amino acids. CFLAR short form (CFLAR-S) includes exon 7 thereby changing the reading frame, creating an early stop codon, and hence a shorter isoform with 221 amino acids. C-FLIP-L is composed of two death effector domains (DEDs) at the amino terminus and a caspase homologous domain, structurally similar to caspase 8 and caspase 10 at carboxy terminus. In contrast C-FLIP-S has two DEDs but lacks caspase homology domain. Presence of rs10190751 regulates the splicing event with rs10190751-A allele resulting in lack of expression of the short form (Fig. 4). In addition to these isoforms recently cFLIP-R forms has been identified in the Raji cells [27]. Due to intronic insertion; CFLAR-R isoform has a premature stop codon resulting in a protein with 212 amino acids and like the CFLAR-S isoform lacks caspase like domain.

Although the characterization of the functional differences of these isoforms is still ongoing, cell type specific pro-apoptotic role of CFLAR-L has been reported. CFLAR-L expression levels are considered critical factor in determining the balance between apoptotic and pro-survival signaling. The CFLAR-L has also been shown to play critical role in autophagy, necroptosis and apoptosis in T-lymphocytes with CFLAR-L deficiency triggering severe cell death upon stimulation [28]. In spite of its major role in regulating death receptor signaling, it has been shown to be involved in regulation of apoptosis by several other mechanism including; modulating the activity of ripoptosome [29] regulation of nectroptosis by preventing caspase 8 activation [30–32], inhibiting autophagosome formation by interfering with conjugation of LC3 and in NFkB signaling with its ectopic expression resulting in NFkB activation [33–35].

Given the important role of CFLAR (CFLIP) as a key inhibitor of processing and activation of caspase 8; its prognostic and therapeutic relevance in AML [36] as well as in development of drug resistance [37] we designed this study to further explore the clinical significance of the CFLAR and its genetic variation especially the splicing SNP (regulating CFLAR-L and CFLAR-S forms) as biomarker of risk of disease as well as with development drug resistance. Our results of siRNA mediated knock down and overexpression of CFLAR in pancreatic cancer cell lines further provides evidence of its involvement in chemo-sensitivity to triptolide.

Gene expression levels of JAK1, AGL, and DTX1 genes, all involved in cell-to cell signaling (Additional file 4: Figure S3) has been associated with triptolide cytotoxicity analysis. JAK1, Janus Kinase 1 is involved in interferon-alpha/beta and -gamma signal transduction pathways and is a critical component of JAK/STAT pathway; AGL is member of 4 alpha-glucanotransferase and is involved in glycogen degradation; DTX1, deltex homolog 1 is involved in NOTCH signaling pathway which is a critical for cell fate determination and has been implicated in several diseases as well as tumorogenesis [38]. In our integrative exploratory analysis we identified several biologically interesting gene-SNP-gene-expression pairs as TIAM1-DTX1, ASXL3: ASCL4, GPATCH2: JAK1, CAMPTA1-CRYGS, ERBB4-NADSYN1 etc.

In recent years there has been significant evidence suggesting triptolide mediated inhibition of ATPase activity of XPB, thereby by influencing transcription as well as Nucleotide excision repair [39]. XPB, also known as ERCC3 is a subunit of transcription factor TFIIH. Triptolide has been shown to influence gene expression by globally reducing gene expression although to not to same extent for all genes by blocking transcription initiation [40, 41]. Antiproliferative effects of triptolide due to inhibition of XPB/TFIIH has also been shown to phenocopy JNK-dependent apoptosis phenotype in Dp53 deficient wing disc cells in Drosophila [42]. This global reduction of transcription caused by triptolide, correlates well with the phenotypes observed in tumour cells and in inflammation. If we take in account these evidences, and if the treatment with triptolide, reduce global transcription, cells with reduction of the CFLAR mRNA isoforms by the splicing SNP will be even more sensitive, since this gene may negatively modulates apoptosis. The KD and overexpression results using Panc-1 cells incubated with triptolide may also be explained in part by taking in account a reduction in global transcription caused by triptolide.

In conclusion, our results identified CFLAR as an important predictor of triptolide cytotoxicity. Splicing SNP-rs10190751 regulates production of CFLAR- long and short isoforms, which are associated with triptolide cytotoxicity. The central role of anti-apoptotic protein c-FLIP (CFLAR product) in regulating death receptor signaling points to the fact that this splicing SNP might of importance to other chemotherapeutic agents. Up-regulation of c-FLIP has been associated with poor clinical outcome and thus could be reliable prognostic factor for several types of cancer, however the significance of CFLAR genetic variation as predictor of therapeutic efficacy has not been explored so far, thus opening up opportunities for future studies.

## Conclusions

Triptolide being an emerging drug, provides us a reason to do a genome wide association study to identify specific genetic polymorphism which may affect triptolide induced cytotoxicity. We observed significant association of triptolide IC50 with SNPs located in biological important genes from apoptotic pathway, such as CFLAR, PPIL3, caspase 8/10, NfKb and STAT6. CFLAR is an upstream regulator of apoptotic pathway. Due to its important position as a regulator of apoptosis, we validated its functional role in triptolide induced cytotoxicity in pancreatic cancer cell line. Our finding shows that CFLAR polymorphism plays important role in cancer cell death induced by triptolide. Further studies are needed to predict the therapeutic response in patients.

## Availability of supporting data

Gene expression data is publically avialble from Gene Expression Omnibus under submission number series: GSE6536. Genotype data is avaiable at HapMap.org-release 23 (www.HapMap.org).

## Additional files

Additional file 1:
**Supplementary Notes.**
Additional file 2: Figure S1. LD plot generated using Haploview for chromosome 2 region flanking CFLAR gene in CEU population. Additional file 3: Figure S2. Genome-wide association study of mRNA expression with Triptolide cytotoxicity. Y-axis represents -Log 10 (P value) and X-axis presents chromosomal location. Additional file 4: Figure S3. Overlapping networks identified by pathway analysis of genes identified in gene expression analysis with Triptolide IC50 in HapMap LCLs using Ingenuity pathway analysis (IPA) tool.

## References

[1] Liu Z, Ma L, Zhou GB (2011). The main anticancer bullets of the Chinese medicinal herb, thunder god vine. Molecules.

[2] Pan J (2010). RNA polymerase - an important molecular target of triptolide in cancer cells. Cancer Lett.

[3] Zhou GS, Hu Z, Fang HT, Zhang FX, Pan XF, Chen XQ (2011). Biologic activity of triptolide in t(8;21) acute myeloid leukemia cells. Leuk Res.

[4] Chen Q, Lu Z, Jin Y, Wu Y, Pan J (2010). Triptolide inhibits Jak2 transcription and induces apoptosis in human myeloproliferative disorder cells bearing Jak2V617F through caspase-3-mediated cleavage of Mcl-1. Cancer Lett.

[5] Miyata Y, Sato T, Ito A (2005). Triptolide, a diterpenoid triepoxide, induces antitumor proliferation via activation of c-Jun NH2-terminal kinase 1 by decreasing phosphatidylinositol 3-kinase activity in human tumor cells. Biochem Biophys Res Commun.

[6] Liu Q, Chen T, Chen H, Zhang M, Li N, Lu Z (2004). Triptolide (PG-490) induces apoptosis of dendritic cells through sequential p38 MAP kinase phosphorylation and caspase 3 activation. Biochem Biophys Res Commun.

[7] Antonoff MB, Chugh R, Skube SJ, Dudeja V, Borja-Cacho D, Clawson KA (2010). Role of Hsp-70 in triptolide-mediated cell death of neuroblastoma. J Surg Res.

[8] Huang M, Zhang H, Liu T, Tian D, Gu L, Zhou M (2013). Triptolide inhibits MDM2 and induces apoptosis in acute lymphoblastic leukemia cells through a p53-independent pathway. Mol Cancer Ther.

[9] Zhao F, Chen Y, Zeng L, Li R, Zeng R, Wen L (2010). Role of triptolide in cell proliferation, cell cycle arrest, apoptosis and histone methylation in multiple myeloma U266 cells. Eur J Pharmacol.

[10] Chugh R, Sangwan V, Patil SP, Dudeja V, Dawra RK, Banerjee S (2012). A preclinical evaluation of Minnelide as a therapeutic agent against pancreatic cancer. Sci Transl Med.

[11] Banerjee S, Thayanithy V, Sangwan V, Mackenzie TN, Saluja AK, Subramanian S (2013). Minnelide reduces tumor burden in preclinical models of osteosarcoma. Cancer Lett.

[12] International HapMap Project. www.hapmap.org.

[13] Wheeler HE, Dolan ME (2012). Lymphoblastoid cell lines in pharmacogenomic discovery and clinical translation. Pharmacogenomics.

[14] Shukla SJ, Dolan ME (2005). Use of CEPH and non-CEPH lymphoblast cell lines in pharmacogenetic studies. Pharmacogenomics.

[15] Stark AL, Dolan ME (2013). Lymphoblastoid cell lines in pharmacogenomics: how applicable are they to clinical outcomes?. Pharmacogenomics.

[16] Jiang J, Fridley BL, Feng Q, Abo RP, Brisbin A, Batzler A (2013). Genome-wide association study for biomarker identification of Rapamycin and Everolimus using a lymphoblastoid cell line system. Front Genet.

[17] Ritz C. SJC: Bioassy Analysis using R. Journal of Statistical Software 2005, 12(5):1-22.

[18] Browning BL, Browning SR (2009). A unified approach to genotype imputation and haplotype-phase inference for large data sets of trios and unrelated individuals. Am J Hum Genet.

[19] Stranger BE, Forrest MS, Dunning M, Ingle CE, Beazley C, Thorne N (2007). Relative impact of nucleotide and copy number variation on gene expression phenotypes. Science.

[20] Barbosa-Morais NL, Dunning MJ, Samarajiwa SA, Darot JF, Ritchie ME, Lynch AG (2010). A re-annotation pipeline for Illumina BeadArrays: improving the interpretation of gene expression data. Nucleic Acids Res.

[21] Huang RS, Duan S, Bleibel WK, Kistner EO, Zhang W, Clark TA (2007). A genome-wide approach to identify genetic variants that contribute to etoposide-induced cytotoxicity. Proc Natl Acad Sci U S A.

[22] Zhuang XM, Liu PX, Zhang YJ, Li CK, Li Y, Wang J, Zhou L, Zhang ZQ. Simultaneous determination of triptolide and its prodrug MC002 in dog blood by LC-MS/MS and its application in pharmacokinetic studies. Journal of ethnopharmacology 2013;150(1):131–7. 10.1016/j.jep.2013.08.018 23994469

[23] Kitzen JJ, de Jonge MJ, Lamers CH, Eskens FA, van der Biessen D, van Doorn L (2009). Phase I dose-escalation study of F60008, a novel apoptosis inducer, in patients with advanced solid tumours. Eur J Cancer.

[24] Yang M, Huang J, Pan HZ, Jin J (2008). Triptolide overcomes dexamethasone resistance and enhanced PS-341-induced apoptosis via PI3k/Akt/NF-kappaB pathways in human multiple myeloma cells. Int J Mol Med.

[25] Carter BZ, Mak DH, Schober WD, McQueen T, Harris D, Estrov Z (2006). Triptolide induces caspase-dependent cell death mediated via the mitochondrial pathway in leukemic cells. Blood.

[26] Miura K, Fujibuchi W, Unno M (2012). Splice variants in apoptotic pathway. Exp Oncol.

[27] Ueffing N, Singh KK, Christians A, Thorns C, Feller AC, Nagl F (2009). A single nucleotide polymorphism determines protein isoform production of the human c-FLIP protein. Blood.

[28] He MX, He YW (2013). CFLAR/c-FLIPL: a star in the autophagy, apoptosis and necroptosis alliance. Autophagy.

[29] Feoktistova M, Geserick P, Kellert B, Dimitrova DP, Langlais C, Hupe M (2011). cIAPs block Ripoptosome formation, a RIP1/caspase-8 containing intracellular cell death complex differentially regulated by cFLIP isoforms. Mol Cell.

[30] Oberst A, Dillon CP, Weinlich R, McCormick LL, Fitzgerald P, Pop C (2011). Catalytic activity of the caspase-8-FLIP(L) complex inhibits RIPK3-dependent necrosis. Nature.

[31] Zhang H, Zhou X, McQuade T, Li J, Chan FK, Zhang J (2011). Functional complementation between FADD and RIP1 in embryos and lymphocytes. Nature.

[32] Kaiser WJ, Upton JW, Long AB, Livingston-Rosanoff D, Daley-Bauer LP, Hakem R (2011). RIP3 mediates the embryonic lethality of caspase-8-deficient mice. Nature.

[33] Chaudhary PM, Eby MT, Jasmin A, Kumar A, Liu L, Hood L (2000). Activation of the NF-kappaB pathway by caspase 8 and its homologs. Oncogene.

[34] Kataoka T, Tschopp J (2004). N-terminal fragment of c-FLIP(L) processed by caspase 8 specifically interacts with TRAF2 and induces activation of the NF-kappaB signaling pathway. Mol Cell Biol.

[35] Kataoka T, Budd RC, Holler N, Thome M, Martinon F, Irmler M (2000). The caspase-8 inhibitor FLIP promotes activation of NF-kappaB and Erk signaling pathways. Current biology : CB.

[36] McLornan D, Hay J, McLaughlin K, Holohan C, Burnett AK, Hills RK (2013). Prognostic and therapeutic relevance of c-FLIP in acute myeloid leukaemia. Br J Haematol.

[37] Lee S, Yoon CY, Byun SS, Lee E, Lee SE (2013). The role of c-FLIP in cisplatin resistance of human bladder cancer cells. J Urol.

[38] Ranganathan P, Weaver KL, Capobianco AJ (2011). Notch signalling in solid tumours: a little bit of everything but not all the time. Nat Rev Cancer.

[39] Chen F, Gao X, Shilatifard A (2015). Stably paused genes revealed through inhibition of transcription initiation by the TFIIH inhibitor triptolide. Genes Dev.

[40] Tee WW, Shen SS, Oksuz O, Narendra V, Reinberg D (2014). Erk1/2 activity promotes chromatin features and RNAPII phosphorylation at developmental promoters in mouse ESCs. Cell.

[41] Villicaña C, Cruz G, Zurita M (2013). The genetic depletion or the triptolide inhibition of TFIIH in p53-deficient cells induces a JNK-dependent cell death in Drosophila. J Cell Sci.

[42] Titov DV, Gilman B, He QL, Bhat S, Low WK, Dang Y (2011). XPB, a subunit of TFIIH, is a target of the natural product triptolide. Nat Chem Biol.

