# Supplementary material for: Genome-wide association analysis identified splicing single nucleotide polymorphism in CFLAR predictive of triptolide chemo-sensitivity
Source: BMC Genomics. 2015 Jun 30;16:483. doi: 10.1186/s12864-015-1614-1 (PMC4485866; doi:10.1186/s12864-015-1614-1)
Supplement: Additional file 1: — Supplementary Notes. [file 12864_2015_1614_MOESM1_ESM.docx]

***Supplementary Notes:***

***Real-Time Quantitative PCR Analysis:*** mRNA expression levels of CFLAR spliced isoforms were quantitated in HAPMAP LCLs and Panc-1 cell lines. Briefly, total RNA was extracted using the RNAeasy kit (QIAGEN, USA) and cDNA was synthesized using High Capacity cDNA reverse transcription kit (Life Technologies, USA). Quantitative real-time PCR was carried out using ABI7900-HT Real-time PCR system and QuantiTect SYBR Green PCR Kit. Gene and isoform specific oligonucleotide primers are listed below.

Common forward Primer 5’- GGACCTTGTGGTTGAGTTGGA-3’ (in exon 5) was used for all CFLAR isoforms

CFLAR –Long isoform reverse primers 5’-GAGCGCCAAGCTGTTCCTTA-3’ (exon 8),

CFLAR- Short isoform reverse primer 5’-CATAGGGTGTTATCATCCTGAAGT-3’ (in exon 7)

CFLAR-Raji isoform reverse primer 5’- TGGGATTCCATATGTTTTCTCCAG-3’ (intron6)

GAPDH, forward primer 5’- AGGGCTGCTTTTAACTCTGGT-3’ and reverse primer 5’- CCCCACTTGATTTTGGAGGGA-3’.

The relative levels of CFLAR isoforms was determined by 2^-ΔΔCT method after normalization to GAPDH levels.

***Association analysis of triptolide cytotoxicity with genetic variation:*** SNP genotype (n= 4098136) data was retrieved from the HapMap (release 23) for all 55 samples (29 female and 26 male). The genomic position of these markers were updated from NCBI build 36 to NCBI build 37 patch 0, based on conversion data from NCBI (<ftp://ftp.ncbi.nih.gov/snp/organisms/human_9606/database/organism_data/b131_SNPChrPosOnRef_37_1.bcp.gz>). In the process of build changes, several SNPs were lost (n=11949). The first quality control measure considered was per SNP call rate, SNPs with a call rate < 0.95 were dropped (n= 599102). Next, an exact test of HWE was conducted and no SNPs where found to have a p-value < 10^-6^, so none were dropped [[18](#_ENREF_18)]. Call rate per sample was calculated and all samples had a rate ≥ 0.95 indicating fairly high quality samples. Finally SNPs with MAF < 0.05 were dropped (n= 1508282), as power would be non-existent to detect an association with the phenotypes. In total 1978803 SNPs in 55 individuals passed genotyping quality control measures. Genotype data for individuals in the 1000 genomes project was retrieved for 29 samples. Since there is no missing data due to the method for calling SNPs, only a MAF filter was used, dropping SNPs with MAF < 0.05. Several SNPs overlap between the 1000 genomes and HapMap data, data obtained from the 1000 genomes project was used preferentially over data obtained from the HapMap. Since not all of the samples used have been sequenced as part of the 1000 genomes project as of current, SNPs in the 1000 genomes project, but not in HapMap (release 23) were imputed for samples not included in the 1000 genomes project.

BEAGLE v3.3.1 [[19](#_ENREF_19)] was used to impute SNPs with the reference of the 1000 genomes Caucasian population (n=87) with SNPs reduced to SNPs with minor allele frequency ≥ 0.01. Due to computational reasons, the imputation was split up into 40 Mb regions on chromosomes with 1 Mb overlap between regions to ensure quality imputation near the edges of the regions (1 Mb overlap region results discarded). The option *lowmem* option was set to “true”, which should not affect results but changes performance characteristics of the BEAGLE program (see documentation). Imputed SNPs were then quality controlled using the dosage R^2^ measure of imputation quality (see BEAGLE documentation), with SNPs removed with a value < 0.3.
