# Supplementary figures and images for: Genome-wide association analysis identified splicing single nucleotide polymorphism in CFLAR predictive of triptolide chemo-sensitivity
Source: BMC Genomics. 2015 Jun 30;16:483. doi: 10.1186/s12864-015-1614-1 (PMC4485866; doi:10.1186/s12864-015-1614-1)

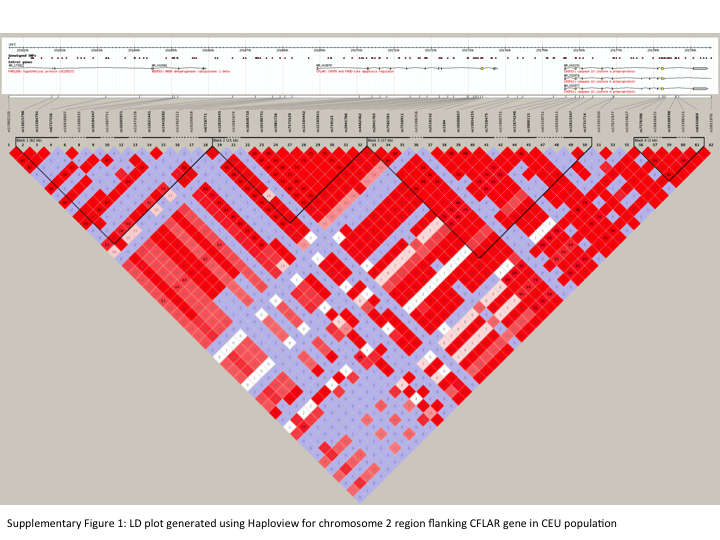

Supplement: Additional file 2: Figure S1. — LD plot generated using Haploview for chromosome 2 region flanking CFLAR gene in CEU population. [file 12864_2015_1614_MOESM2_ESM.tiff]

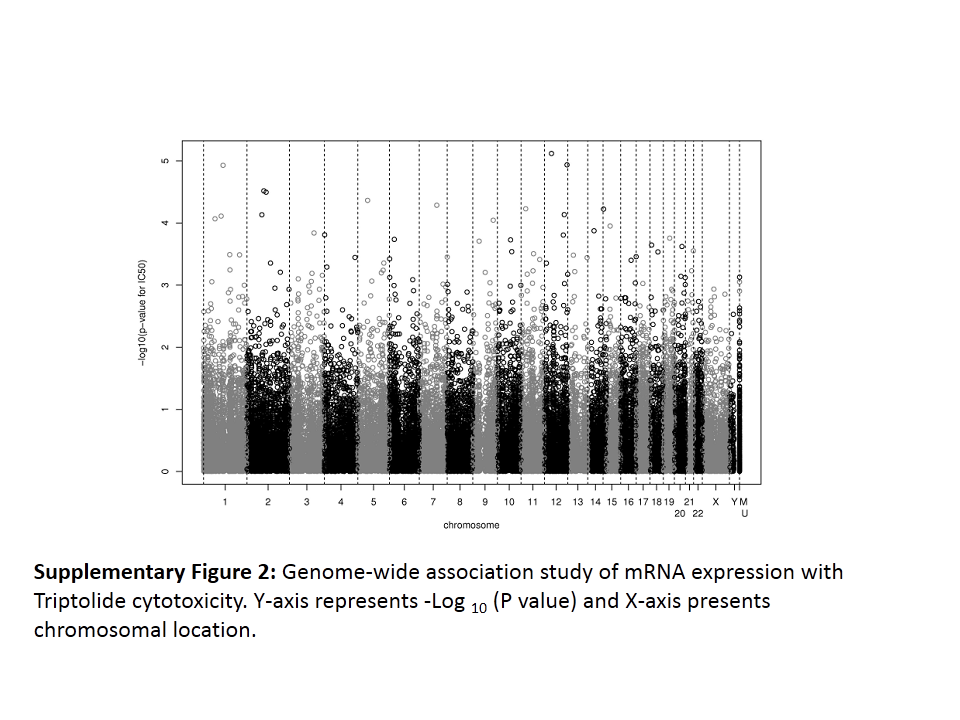

Supplement: Additional file 3: Figure S2. — Genome-wide association study of mRNA expression with Triptolide cytotoxicity. Y-axis represents -Log 10 (P value) and X-axis presents chromosomal location. [file 12864_2015_1614_MOESM3_ESM.tiff]

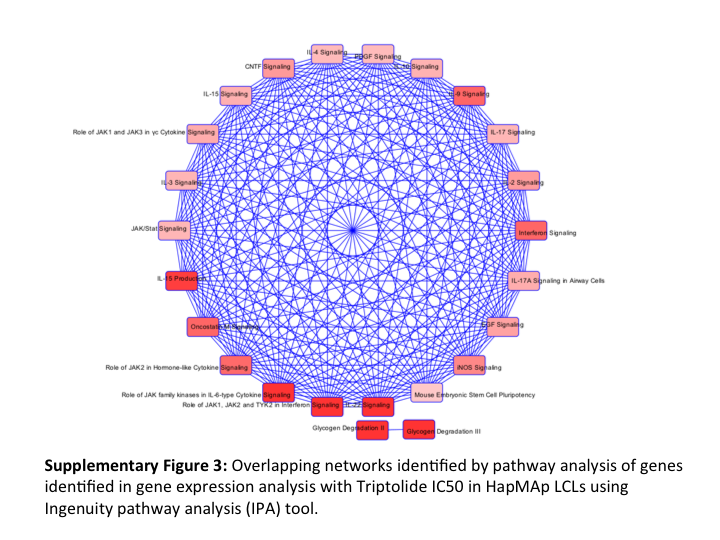

Supplement: Additional file 4: Figure S3. — Overlapping networks identified by pathway analysis of genes identified in gene expression analysis with Triptolide IC50 in HapMap LCLs using Ingenuity pathway analysis (IPA) tool. [file 12864_2015_1614_MOESM4_ESM.tiff]
